# Bone mass density following developmental exposures to perfluoroalkyl substances (PFAS): a longitudinal cohort study

**DOI:** 10.1186/s12940-022-00929-w

**Published:** 2022-11-19

**Authors:** Annelise Blomberg, Jann Mortensen, Pál Weihe, Philippe Grandjean

**Affiliations:** 1 Department of Environmental Health, Harvard T.H. Chan School of Public Health, Boston, MA USA; 2 Division of Occupational and Environmental Medicine, Lund University, Scheelevägen 2, 22363 Lund, Sweden; 3 Department of Clinical Physiology and Nuclear Medicine, Rigshospitalet, Copenhagen University Hospital, Copenhagen, Denmark; 4 Department of Medicine, The Faroese National Hospital, Torshavn, Faroe Islands; 5 Department of Occupational Medicine and Public Health, Faroese Hospital System, Torshavn, Faroe Islands; 6 Center of Health Science, University of the Faroe Islands, Torshavn, Faroe Islands; 7 Department of Environmental Medicine, University of Southern Denmark, Odense, Denmark

**Keywords:** Per- and polyfluoroalkyl substances, PFAS, DXA, Childhood, Bone mass density

## Abstract

**Background:**

Environmental exposures to industrial chemicals, including perfluoroalkyl substances (PFAS), may play a role in bone development and future risk of osteoporosis. However, as prospective evidence is limited, the role of developmental PFAS exposures in bone density changes in childhood is unclear. The objective of this study was to estimate associations between serum-PFAS concentrations measured in infancy and early childhood and areal bone mineral density (aBMD) measured at age 9 years in a birth cohort of children from the Faroe Islands.

**Methods:**

We prospectively measured concentrations of five PFAS in cord serum and serum collected at 18 months, 5 years and 9 years, and conducted whole-body DXA scans at the 9-year clinical visit. Our study included 366 mother-child pairs with DXA scans and at least one PFAS measurement. We estimated covariate-adjusted associations of individual PFAS concentrations with age-, sex- and height-adjusted aBMD *z-*scores using multivariable regression models and applied formal mediation analysis to estimate the possible impact of by several measures of body composition. We also evaluated whether associations were modified by child sex.

**Results:**

We found PFAS exposures in childhood to be negatively associated with aBMD z-scores, with the strongest association seen for perfluorononanoic acid (PFNA) at age 5 years. A doubling in age-5 PFNA was associated with a 0.15 decrease in aBMD z-score (95% CI: − 0.26, − 0.039). The PFNA-aBMD association was significantly stronger in males than females, although effect modification by sex was not significant for other PFAS exposures. Results from the mediation analysis suggested that any potential associations between aBMD and 18-month PFAS concentrations may be mediated by total body fat and BMI, although most estimated total effects for PFAS exposures at age 18 months were non-significant. PFAS exposures at age 9 were not associated with age-9 aBMD *z*-scores.

**Conclusions:**

The PFAS-aBMD associations identified in this and previous studies suggest that bone may be a target tissue for PFAS. Pediatric bone density has been demonstrated to strongly track through young adulthood and possibly beyond; therefore, these prospective results may have important public health implications.

**Supplementary Information:**

The online version contains supplementary material available at 10.1186/s12940-022-00929-w.

## Introduction

Perfluoroalkyl substances (PFAS) constitute a class of synthetic perfluorinated chemicals that are widespread in the environment and detectable in most human blood samples [1]. PFAS have been detected in human bone [2, 3], and toxicological studies suggest that PFAS exposure may alter bone ossification and accrual [4, 5]. Several cross-sectional epidemiological studies in adults have found associations between PFAS exposures and measures of bone health [6–10], and one study also found an association between baseline plasma PFAS concentrations and reductions in bone mass density over a 2-year period in overweight adults [8].

Given this evidence, the question arises if PFAS may induce changes in bone structure beginning in childhood. Bone mass accrues through childhood and adolescence until peak bone mass is reached by early adulthood [11]. This peak bone mass is an important determinant of future risk of osteoporosis [12, 13]. Bone development in infancy and childhood may be particularly vulnerable to chemical disruption. Because PFAS are endocrine disruptors, developmental PFAS exposures may alter the hormonal regulation of skeletal formation and the bone remodeling process and ultimately impact peak bone mass [14].

Five epidemiological studies to date have investigated a possible association between PFAS exposures and bone density in childhood and adolescence. Two cross-sectional studies evaluated associations at approximately 8 years of age. While the smaller of these studies only found suggestive inverse associations [15], the larger study reported that higher concentrations of PFOA, PFOS and the overall PFAS mixture were associated with lower areal bone mineral density (aBMD) z-scores [16]. A third cross-sectional study in NHANES adolescents ages 12–19 years found that PFOA and the overall PFAS mixture were similarly associated with lower aBMD z-scores in males but not females [17]. In addition, two prospective studies evaluated associations between maternal serum concentrations collected during pregnancy and indicators of bone health in older childhood [18] and adolescence [19], and identified some inverse associations.

However, each of these studies only included PFAS concentrations measured at one point in time (either prenatally or in mid-childhood), and therefore were unable to evaluate whether specific periods of bone development may be more susceptible to PFAS exposure. In addition, these studies differed in how they accounted for the role of body composition in a possible PFAS-bone association. While one study adjusted for height and lean body mass [19] and three others used height-adjusted aBMD z-scores as their primary outcome [16–18], one did not adjust for any measure of body composition, arguing that it may be on the causal pathway [15]. This connection is important because previous research has demonstrated that body composition, including fat mass, is an important determinant of childhood bone mass [20, 21]. Furthermore, several studies have also identified associations between PFAS exposures and childhood adiposity [22–24]. Mediation analysis can be used to formally investigate potential influence by body composition and may yield insight into biological mechanisms underlying any observed associations.

To address the possible impact of developmental PFAS exposures on skeletal development during childhood, we studied the associations of repeated measures of serum-PFAS concentrations with aBMD at age 9 years in a birth cohort of children from the Faroe Islands, a community with wide ranges of PFAS exposures and fairly uniform social conditions and genetic background [25]. We estimated the total effect of PFAS exposure on aBMD and used a formal mediation analysis to assess the possible impact of body composition. We hypothesized that PFAS exposures would be associated with reduced bone mass density, but that part of this total effect might be mediated by changes in body composition during childhood growth.

## Methods

### Study population and design

The Faroese Cohort 5 includes 490 full-term singleton children and their mothers. Participants were consecutively recruited at the National Hospital in Tórshavn, Faroe Islands between October 2007 and April 2009 [26, 27]. A pregnancy questionnaire included questions on medical history, current health, diet, and social factors before and during pregnancy. Obstetric information on parity and pre-pregnancy weight and height were extracted from medical records. We collected umbilical cord blood samples immediately after birth. The cohort children were then invited for follow-up visits at ages 18 months, 5 years and 9 years. Each follow-up included a clinical examination, measurements of height and weight, blood sampling, and completion of a standard questionnaire. At 18 months, the questionnaire included questions about the duration of breastfeeding, and at 9 years the questionnaire included questions about diet and physical activity (estimated as the average number of non-school hours per week spent exercising or playing). A study selection flow chart is included in the Supplementary Material as Fig. S1. This study included a total of 366 participants with the necessary exposure, outcome, and covariate information.

The Harvard T.H. Chan institutional review board and Faroese ethical review committee approved the study protocol, and written informed consent was obtained from all mothers.

### Exposure assessment

We measured concentrations of five major PFAS compounds (PFOS, PFOA, perfluorononanoic acid (PFNA), perfluorohexanesulfonic acid (PFHxS), and perfluorodecanoic acid (PFDA)) in serum obtained from cord blood at birth and at the 18-month, 5-year and 9-year clinical examinations. Samples were frozen shortly after separation and stored at − 80 °C. PFAS concentrations were measured using online solid-phase extraction followed by high-pressure liquid chromatography with tandem mass spectrometry (LC-MS/MS) [28] at the Unit of Environmental Medicine, Institute of Public Health, University of Southern Denmark. All PFAS had a limit of detection (LOD) of 0.03 ng/ml. Samples with concentrations below the LOD were assigned a concentration of 0.015 ng/ml. Measurement imprecision was assessed by within-batch and between-batch coefficients of variation, which were < 3% and 5–6% respectively. The laboratory regularly participates in the German External Quality Assessment Scheme (G-EQUAS), organized by the German Society of Occupational Medicine, to ensure quality at a high level.

### Outcome assessment

We conducted whole-body DXA scans of subjects at the 9-year clinical visit using the Norland XR-800 system (Norland, Fort Atkinson WI, USA). Total body fat and lean mass were also measured in the same DXA scans. The system was calibrated daily. Quality control was performed using a QA Calibration Standard and a QC Phantom (BMD: 0.867 g/cm^2^; fat mass: 437.89 g; lean mass: 604.71 g) (Norland, Fort Atkinson WI, USA). Whole body scan precision (coefficient of variation (CV)) for BMC, aBMD and area was 0.67, 0.78 and 0.66%, respectively. The CV for lean mass and fat mass was 0.93 and 1.4%. Data were analyzed using the Illuminatus DXA software (Norland, Fort Atkinson WI, USA). We then calculated aBMD excluding the head (total body less head, TBLH) and used a reference dataset to derive age-, sex-, and height-adjusted TBLH aBMD *z*-scores (hereafter referred to as aBMD *z*-scores) [29], as is recommended by the International Society for Clinical Densitometry (ISCD) [30].

### Statistical analyses

We estimated the total associations between individual serum PFAS concentrations and aBMD *z*-scores using multivariable regression models. PFAS concentrations were natural log-transformed to minimize the impact of outlier values. The outcome, aBMD *z*-score, was normally distributed and modeled as a continuous variable. Associations are reported as the expected difference in aBMD *z*-score for a doubling in the serum-PFAS concentration.

**Fig. 1:**
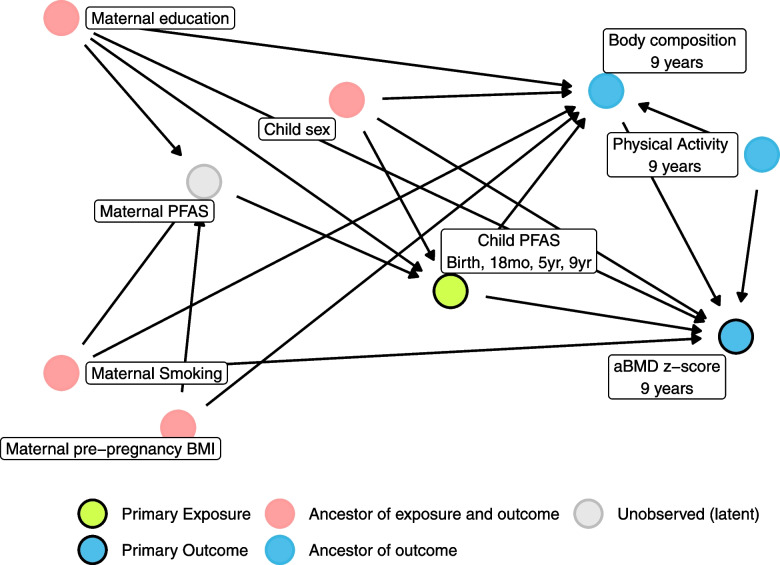
DAG for PFAS exposures at 9 years

Models were adjusted a priori for covariates that have been associated with maternal or child PFAS concentrations in previous studies [31–36] and may also be associated with aBMD [37–39]. All models were adjusted for child sex, maternal smoking during pregnancy (yes, no), maternal pre-pregnancy BMI (kg/m^2^), parity (primiparous, multiparous), and maternal education (low, medium, high) as a proxy for socioeconomic status. Because we were modeling the total association between PFAS and aBMD z-score, we did not adjust for any measures of body composition, which were on the hypothesized causal path. The directed acyclic graph (DAG) for our models is included as Fig. 1. We evaluated possible effect modification by sex in a second set of models that included an interaction term between sex and PFAS concentration. We used these interaction models to estimate sex-specific PFAS associations and test whether differences by sex were significant (p-interaction < 0.1).

We also estimated the controlled direct effect of PFAS on aBMD *z*-score by adjusting each model for age-9 total fat mass (g), total lean mass (g), and BMI (kg/m^2^) separately. The direct effect models were also adjusted for physical activity (hours/week), which may be associated with body composition and bone density [40].

#### Sensitivity analyses

We conducted several analyses to assess whether our estimates of total association were sensitive to model specification. First, we added an additional adjustment for exclusive breastfeeding duration (months) to our models of PFAS exposure at age 18 months, 5 years and 9 years, as breastfeeding is an important PFAS exposure source [41] and may be associated with aBMD [39]. Second, we assessed whether our primary aBMD *z*-scores were sensitive to bias by using a different set of age- and sex-adjusted reference values for total aBMD (not excluding head) specific to the Norland XR system [42]. Third, we allowed for nonlinear associations between PFAS and aBMD by fitting generalized additive models (GAMs) with penalized thin-plate splines for each PFAS term, and compared the fit of these non-linear models to their linear counterparts using Akaike Information Criterion (AIC) values [43].

#### Mediation analysis

We conducted a formal average causal mediation analysis to investigate whether differences in body composition (total body fat, total lean mass, and BMI) at age 9 years mediated the observed associations between PFAS (measured at birth, 18 months, and 5 years) and age-9 aBMD *z*-scores. First, we tested whether the PFAS exposure was significantly (*p*-value < 0.05) associated with the mediator, and whether the mediator was significantly associated with aBMD *z*-score after adjustment for PFAS exposure. Each potential mediation model was evaluated only if these two criteria were met [44]. We then decomposed the total PFAS effect (TE) into two components:

1. The average causal mediated effect (ACME), representing the estimated average change in aBMD that is mediated through changes in body composition; and
2. The average direct effect (ADE), representing the average estimated effect of PFAS exposure on aBMD that is not mediated through changes in body composition.

The percent mediated (PM) was calculated as the ratio of the mediated effect and the total effect (ACME/TE) × 100%. All mediation models were adjusted for the covariates included in our primary models, as well as for weekly physical activity, which may act as a confounder in the association between body composition and aBMD (Fig. 1). Confidence intervals were calculated using nonparametric bootstrapping (*n* = 1000) [45, 46].

Our primary mediation analysis was limited to mediators measured at age 9, the only time when DXA scans were conducted in this cohort. To address this limitation, we conducted an additional sensitivity analysis to better represent the assumed temporality underlying our mediation analysis. We examined whether BMI measured at age 5 mediated observed associations between PFAS (measured at birth and 18 months) and aBMD *z*-scores.

All statistical analyses were conducted in R version 4.2.1 (2022-06-23) [47]. DAGs were drawn using the packages “dagitty” version 0.3.1 and “ggdag” version 0.2.6 [48, 49]. Causal mediation analyses were completed using the mediation package version 4.5.0 [46].

## Results

A total of 366 participants (75%) from the original birth cohort had DXA measurements at age 9, PFAS concentrations measured from one or more visits, and complete covariate information (Fig. S1 and Table S1). Children with age-9 DXA scans were generally similar to the overall study population (Table S2), although there was a significant difference in maternal education where children with a DXA scan had a higher percentage of mothers with a medium-level education, while children without a DXA scan had a higher percentage of mothers with either a low or high education level. Maternal and child characteristics for this population are shown in Table 1. Our study population was 52% male, and most children had older siblings (72%). The average age at the DXA visit was 9.07 years (SD: 0.14). Population characteristics were similar in females and males, except that males reported higher physical activity at age 9 years (Table S3). Population characteristics were also similar across the subsets of study participants with measured PFAS concentrations at each time point (Table S4).

**Table 1:** Characteristics of the Faroese mother-child pairs

Characteristic	Level	N (%) or Mean (SD)
N		366
Sex	Female	177 (48.4)
	Male	189 (51.6)
Maternal education	Low	117 (32.0)
	Medium	103 (28.1)
	High	146 (39.9)
Maternal smoking	Yes	60 (16.4)
	No	306 (83.6)
Parity	Primiparous	104 (28.4)
	Multiparous	262 (71.6)
Maternal pre-pregnancy BMI (kg/m^2^)		24.41 (4.48)
Weekly physical activity, hours	< 1	86 (23.5)
	2–3	199 (54.4)
	> 4	81 (22.1)

Serum-PFAS concentrations were above the LOD in 99% of the serum samples. Concentrations of PFOA and PFOS showed a maximum at 18 months, while concentrations of PFDA, PFHxS and PFNA were the highest at age 5 years (Fig. S2 and Table S5). Cord blood serum concentrations at birth varied significantly by some population characteristics. For example, first-born children had higher PFAS concentrations for all measured PFAS compounds, and children born to mothers who reported smoking during pregnancy had higher concentrations of PFOA and PFHxS (Table S6).

Concentrations of different PFAS compounds were correlated within visit. For example, PFNA and PFDA showed a correlation of 0.85 at birth, 0.89 at 18 months, and 0.87 at 9 years. Concentrations of individual PFAS compounds were also correlated across time. For example, PFOS at age 9 had a correlation of 0.81 with PFOS at age 5 and 0.81 with PFOS at 18 months (Fig. S3).

**Fig. 2:**
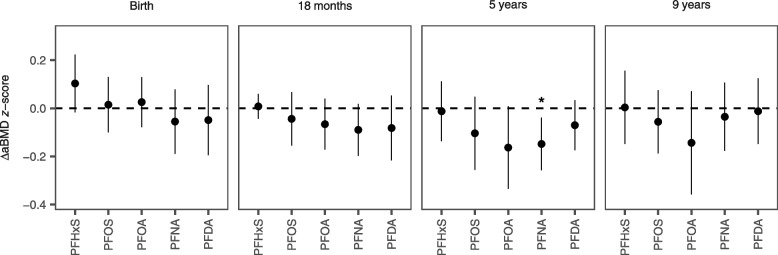
Associations between aBMD and PFAS concentrations at birth, 18 months, age 5 and age 9

Higher concentrations of PFOA and PFNA at age 5 years were associated with decreased aBMD *z*-scores at age 9 (Fig. 2 and Table S7). A doubling in age-5 PFNA concentrations was associated with a − 0.15 decrease in aBMD z-score (95% CI: − 0.26, − 0.039), while a doubling in age-5 PFOA concentrations was associated with a − 0.16 decrease in aBMD *z*-score (95% CI: − 0.33, 0.0086). PFNA concentrations measured at age 18 months were also marginally associated with decreased aBMD *z*-scores at age 9, which other PFAS concentrations at age 18 months were consistently but non-significantly associated with aBMD *z*-scores. We did not find any associations between PFAS concentrations measured at birth or at age 9 and aBMD *z-*scores. Effect modification by sex was marginally significant for PFNA exposures at age 5 years (p-interaction = 0.11), where the effect in males was stronger than in females (Fig. S4). Otherwise, effect modification by sex was non-significant.

When we estimated the controlled direct effect of PFAS exposures on aBMD *z-*scores by adjusting for BMI and total fat mass, negative PFAS-aBMD associations were attenuated, especially for 18-month PFAS exposures where the estimated direct effect was near zero. The direct associations after adjustment for total lean mass were generally more similar to the estimated total associations (Fig. S5).

When we further adjusted our primary models for the duration of exclusive breastfeeding, the estimated associations remained consistent with our primary results (Fig. S6). In our second sensitivity analysis using age- and sex-adjusted aBMD *z*-scores rather than age-, sex-, and height-adjusted aBMD *z*-scores, we found stronger negative associations between PFAS exposures at age 5 years and aBMD z-scores (Fig. S7). Finally, most linear models (65%) fit better than non-linear models when compared using AIC values. Of the seven models that fit better with non-linear splines, only one showed a significant effect of non-linear PFAS (PFOA exposure at age 18 months). However, the fit appeared linear across most of the distribution of serum-PFAS concentrations (Fig. S8).

We evaluated whether body composition could mediate the effect of PFAS on aBMD using three measures of body composition measured at age 9 years, i.e., BMI, total body fat and total lean mass. Of the possible mediation models considered, BMI was significantly associated with 18-month PFDA, PFNA, and PFOA concentrations, as well as 5-year PFOA concentrations. Total body fat was associated with the same exposures, as well as 18-month PFOS concentrations. Total lean mass was not associated with any PFAS exposures. BMI measured at age 5 years was associated with 18-month PFDA and PFNA concentrations. All measures of body composition were associated with aBMD at age 9.

**Fig. 3:**
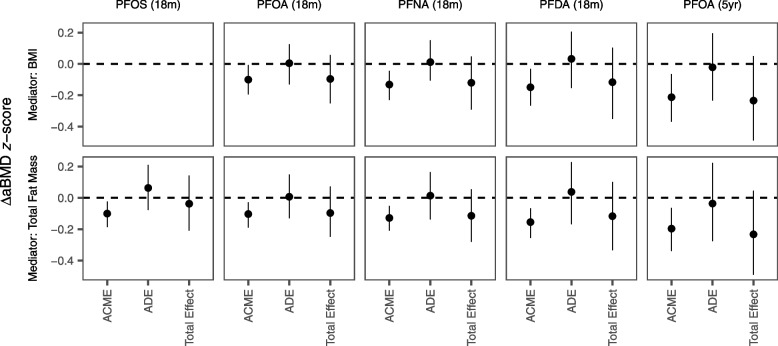
Mediation analysis of the PFAS-aBMD association by BMI and total body fat

The mediation models generally showed significant ACMEs by BMI and total fat mass between 18-month PFAS and aBMD z-scores, but the total effects for most models were non-significant (Fig. 3 and Table S8). This suggests that any potential associations between 18-month PFAS concentrations and aBMD *z*-scores may be mediated by body composition. When we evaluated mediation by age-5 BMI for PFNA and PFDA exposures at age 18 months, we found significant ACMEs for both models, as well as a significant total effect for PFNA on aBMD z-score (Fig. S9), where 49% of the total effect of 18-month PFNA on aBMD z-score was mediated by BMI at age 5.

## Discussion

This study measured bone mineral density in 366 children at age 9 years and assessed possible associations with PFAS exposures both prenatally and in early- and mid-childhood. We found that age-, sex- and height-adjusted aBMD *z*-scores showed consistent negative associations with PFAS exposures at 18 months and 5 years, with the strongest effects found for PFNA and PFOA exposures at age 5. Differences between our primary model using age-, sex-, and height-adjusted aBMD *z*-scores and results from a sensitivity analysis using only age- and sex-adjusted *z*-scores indicate the importance of accounting for height as a possible confounder, especially given the known close relationship between prepuberty bone mass and height [13, 50].

We hypothesized that any PFAS-aBMD association may be mediated by body composition, specifically BMI, total body fat, and/or lean mass, which have been associated with PFAS exposures both in the present cohort and in other studies [22–24, 51]. Results from our mediation models suggest that 18-month PFAS exposures may affect both aBMD and body composition, although the lack of a strong total effect limits the conclusions that can be drawn from this analysis. In contrast, age-5 PFAS exposures were generally not associated with body composition. While the present study does not explore the possible pathogenesis of decreased bone mineral content, our results may indicate that different mechanisms may be in operation for PFAS exposures at different developmental stages.

These findings are meaningful in the light of experimental evidence. Reduced ossification occurred in neonatal mice at low PFOA doses that did not affect fetal weight, suggesting that ossification abnormalities may represent a specific toxicity(s) rather than developmental delays [52]. PFAS act as peroxisome proliferator-activated receptor (PPAR) agonists, which are expressed in osteoclasts and osteoblasts and may impact osteogenic differentiation [2, 5, 53, 54]. Furthermore, PFAS may inhibit enzyme activity on androgen secretion pathways [55] or act as androgen receptor antagonists [7, 56], which may impact bone formation. A recent study found that PFOA may act as an antagonist on the Vitamin D receptor [4], possibly contributing to a functional vitamin D deficiency. Taken together, this evidence indicates that PFAS exposures could affect bone ossification via several biological mechanisms. Given this background, the U.S. Agency for Toxic Substances and Disease Registry chose the experimental data on skeletal toxicity [5] as the criterion for identifying a Minimal Risk Level for PFOA [57].

Our results add to several previous studies that also found associations between developmental PFAS exposures and bone health in children. Two longitudinal studies have evaluated associations between prenatal PFAS exposures and indicators of childhood and adolescence bone health. One found an inverse association between prenatal PFOS exposure and BMD measured at age 17 years, although the association was null after adjustment for height [19]. The second study found inverse associations between prenatal PFOA and PFNA and age-12 BMC z-scores for the hip and forearm, but no associations with TBLH age-, sex-, race- and height-adjusted BMC and aBMD *z*-scores [18]. In comparison, our study did not find an association between any prenatal PFAS exposures and age-9 aBMD *z*-scores. However, both previous studies estimated prenatal exposures using maternal serum-PFAS concentrations from pregnancy, while we assessed prenatal exposures using cord serum-PFAS concentrations from delivery. Although closely correlated, these different exposure measurements are not entirely comparable because maternal serum concentrations may change during the course of pregnancy and not only reflect prenatal exposures, but may also contribute to infancy exposures via transfer by breastmilk [41, 58].

Three additional studies evaluated cross-sectional associations in childhood. In a small pilot study of children aged 8–12 (*n* = 48), PFAS were associated with non-significant decreases in several parameters of bone health, with the strongest associations found for PFNA [15]. A larger cross-sectional study of children (*n* = 576, mean age = 8) similarly found that higher plasma-PFAS concentrations were associated with lower aBMD age-, sex-, and height-adjusted z-scores, with the strongest and most consistent association found for PFOA [16]. Finally, a third cross-sectional study of adolescents ages 12–19 years using data from NHANES 2011–2016 (*n* = 848) found that an increase in PFOA and the overall PFAS mixture was associated with a decrease in age-, sex-, and height-adjusted aBMD *z-*scores in males but not females [17]. Compared to these cross-sectional studies, we found stronger associations between aBMD and earlier childhood serum-PFAS concentrations measured at ages 18 months and 5 years. Our study is the first to examine PFAS exposures in these early years, which may reflect important developmental windows in regard to programming of bone development. Thus, despite the fairly long half-lives of PFAS, identification of the most vulnerable time windows likely requires assessment of PFAS concentrations at different ages.

In regard to limitations, our primary models or mediation models may be potentially impacted by residual confounding (e.g., by socioeconomic status or diet). Given the homogeneity of the Faroese population [25], this concern is probably minor. Our mediation model may also be biased by confounding of the association between body composition and bone density (so-called collider-stratification bias). However, we were able to control for exercise, an important potential cofactor in this regard. Pubertal status is an unlikely confounder as all our measurements were conducted at age 9 years, i.e., pre-puberty in most children. Finally, our mediation analyses were only able to evaluate mediation by total fat mass and total lean mass measured at the same time as the primary outcome, making it difficult to distinguish whether body composition is really acting as a mediator. To address this, however, we were able to evaluate whether BMI measured at age-5 was a possible mediator, better representing the temporality assumed in the mediation models [59].

## Conclusions

Our study examines prospective associations between childhood aBMD and PFAS exposure profiles measured longitudinally across childhood development. We assessed pediatric bone density using the recommended measurement of age-, sex-, and height-adjusted TBLH aBMD *z-*score. Pediatric bone density has been shown to strongly track from childhood through young adulthood and predict peak bone mass [11, 60–62]. Peak bone mass, in turn, is an important determinant of future risk of osteoporosis [12, 13]. While further studies are needed to evaluate associations of PFAS exposures with clinical outcomes like fractures, the consistent associations found in this and previous studies support the possibility that bone may be a target tissue for developmental PFAS toxicity in humans.

## Supplementary Information

**Additional file 1: Table S1.** Count of Missing Covariates. **Table S2.** Baseline covariates for mother-child pairs in the Faroe Island Cohort 5, stratified by whether or not the child completed a DXA scan at age 9. **Table S3.** Baseline covariates of study participants, stratified by sex. **Table S4.** Baseline covariates of study participants, stratified by the time point of PFAS exposure measurement. **Table S5.** PFAS concentrations (ng/ml) by visit. **Table S6.** PFAS concentrations at birth (ng/ml) by study covariates. **Table S7.** Estimated total association between serum PFAS concentrations and aBMD at age 9, presented as the expected diference in aBMD for a doubling in serum PFAS. **Table S8.** Estimated ADE and ACME from causal mediation models. **Fig. S1.** Study selection flow chart. **Fig. S2.** Distribution of serum PFAS concentrations by visit. **Fig. S3.** Correlation of serum PFAS concentrations. **Fig. S4.** Associations between PFAS exposures measured at birth, 18 months, age 5 and age 9 and aBMD z-scores measured at age 9 by sex. **Fig. S5.** Direct and total associations between PFAS exposures measured at birth, 18 months, age 5, and age 9 and aBMD z-scores measured at age 9. **Fig. S6.** Associations between PFAS exposures measured at 18 months, and age 5 and 9 years and aBMD z-scores measured at age 9 years, with and without additional adjustment for duration of exclusive breastfeeding. **Fig. S7.** Comparison of associations between PFAS and age-, sex- and height-adjusted aBMD z-scores (primary outcome) vs. age- and sex-adjusted aBMD z-scores. **Fig. S8.** Estimated thin-plate splines for significant nonlinear PFAS terms. **Fig. S9.** Mediation of the association between 18-month PFNA and PFDA concentrations and aBMD z-scores by BMI measured at age 5 years, limited to the 244 study participants who have 18-month PFAS measurements, 9-year DXA scans, complete covariate information and BMI values measured at age 5 years.

## Data Availability

The Faroe Island Cohort 5 data are available to collaborating scientists following strict data privacy protocols, in respect of the GDPR.

## Abbreviations

aBMD: Areal bone mineral density
PFAS: Perfluoroalkyl substance
PFOS: Perfluorooctanesulfonic acid
PFOA: Perfluorooctanoic acid
PFHxS: Perfluorohexanesulfonic acid
PFNA: Perfluorononanoic acid
PFDA: Perfluorodecanoic acid
TBLH: Total body less head
DAG: Directed acyclic graph
TE: Total effect
ADE: Average direct effect
ACME: Average causal mediated effect
