# Correction: Bone mass density following developmental exposures to perfluoroalkyl substances (PFAS): a longitudinal cohort study

**DOI:** 10.1186/s12940-023-00968-x

**Published:** 2023-01-30

**Authors:** Annelise Blomberg, Jann Mortensen, Pal Weihe, Philippe Grandjean

**Affiliations:** 1 Department of Environmental Health, Harvard T.H. Chan School of Public Health, Boston, MA USA; 2 Division of Occupational and Environmental Medicine, Lund University, Scheelevägen 2, 22363 Lund, Sweden; 3 Department of Clinical Physiology and Nuclear Medicine, Rigshospitalet, Copenhagen University Hospital, Copenhagen, Denmark; 4 Department of Medicine, The Faroese National Hospital, Torshavn, Faroe Islands; 5 Department of Occupational Medicine and Public Health, Faroese Hospital System, Torshavn, Faroe Islands; 6 Center of Health Science, University of the Faroe Islands, Torshavn, Faroe Islands; 7 Department of Environmental Medicine, University of Southern Denmark, Odense, Denmark

**Correction: Environ Health 21, 113 (2022)**

**https://doi.org/10.1186/s12940-022-00929-w**

Following publication of the original article [1], the affiliation details for Philippe Grandjean were incorrectly given as '2,7' but should have been '1,7'.

This has been corrected in this correction article and the original article has been updated.
