# Supplementary material for: Bone mass density following developmental exposures to perfluoroalkyl substances (PFAS): a longitudinal cohort study
Source: Environ Health. 2022 Nov 19;21:113. doi: 10.1186/s12940-022-00929-w (PMC9675242; doi:10.1186/s12940-022-00929-w)
Supplement: Supplementary file 1 — Additional file 1: Table S1. Count of Missing Covariates. Table S2. Baseline covariates for mother-child pairs in the Faroe Island Cohort 5, stratified by whether or not the child completed a DXA scan at age 9. Table S3. Baseline covariates of study participants, stratified by sex. Table S4. Baseline covariates of study participants, stratified by the time point of PFAS exposure measurement. Table S5. PFAS concentrations (ng/ml) by visit. Table S6. PFAS concentrations at birth (ng/ml) by study covariates. Table S7. Estimated total association between serum PFAS concentrations and aBMD at age 9, presented as the expected diference in aBMD for a doubling in serum PFAS. Table S8. Estimated ADE and ACME from causal mediation models. Fig. S1. Study selection flow chart. Fig. S2. Distribution of serum PFAS concentrations by visit. Fig. S3. Correlation of serum PFAS concentrations. Fig. S4. Associations between PFAS exposures measured at birth, 18 months, age 5 and age 9 and aBMD z-scores measured at age 9 by sex. Fig. S5. Direct and total associations between PFAS exposures measured at birth, 18 months, age 5, and age 9 and aBMD z-scores measured at age 9. Fig. S6. Associations between PFAS exposures measured at 18 months, and age 5 and 9 years and aBMD z-scores measured at age 9 years, with and without additional adjustment for duration of exclusive breastfeeding. Fig. S7. Comparison of associations between PFAS and age-, sex- and height-adjusted aBMD z-scores (primary outcome) vs. age- and sex-adjusted aBMD z-scores. Fig. S8. Estimated thin-plate splines for significant nonlinear PFAS terms. Fig. S9. Mediation of the association between 18-month PFNA and PFDA concentrations and aBMD z-scores by BMI measured at age 5 years, limited to the 244 study participants who have 18-month PFAS measurements, 9-year DXA scans, complete covariate information and BMI values measured at age 5 years. [file 12940_2022_929_MOESM1_ESM.pdf]

Supplemental Material

Bone Mass Density following Developmental Exposures to Perfluoroalkyl Substances

Annelise Blomberg, Jann Mortensen, Pál Weihe, Philippe Grandjean

September 13, 2022

List of Tables

S1 Count of Missing Covariates 2
S2 Baseline covariates for mother-child pairs in the Faroe Island Cohort 5, stratified by whether or not the child completed a DXA scan at age 9. 2
S3 Baseline covariates of study participants, stratified by sex 3
S4 Baseline covariates of study participants, stratified by the time point of PFAS exposure measurement 4
S5 PFAS concentrations (ng/ml) by visit 5
S6 PFAS concentrations at birth (ng/ml) by study covariates 6
S7 Estimated total association between serum PFAS concentrations and aBMD at age 9, presented as the expected diference in aBMD for a doubling in serum PFAS. 8
S8 Estimated ADE and ACME from causal mediation models. 9

List of Figures

S1 Study selection flow chart 11
S2 Distribution of serum PFAS concentrations by visit 12
S3 Correlation of serum PFAS concentrations 13
S4 Associations between PFAS exposures measured at birth, 18 months, age 5 and age 9 and aBMD z-scores measured at age 9 by sex 14
S5 Direct and total associations between PFAS exposures measured at birth, 18 months, age 5, and age 9 and aBMD z-scores measured at age 9 14
S6 Associations between PFAS exposures measured at 18 months, and age 5 and 9 years and aBMD z-scores measured at age 9 years, with and without additional adjustment for duration of exclusive breastfeeding 15
S7 Comparison of associations between PFAS and age-, sex- and height-adjusted aBMD z-scores (primary outcome) vs. age- and sex-adjusted aBMD z-scores 15
S8 Estimated thin-plate splines for significant nonlinear PFAS terms 16
S9 Mediation of the association between 18-month PFNA and PFDA concentrations and aBMD z-scores by BMI measured at age 5 years, limited to the 244 study participants who have 18-month PFAS measurements, 9-year DXA scans, complete covariate information and BMI values measured at age 5 years. 16

1 Supplemental Tables

Table S1: Count of Missing Covariates

Characteristic	N (%)
Total N	379 (100)
Missing at least one covariate	13 (3.4)
Sex	0 (0)
Maternal education	9 (1.8)
Maternal smoking	2 (0.41)
Parity	2 (0.41)
Maternal BMI	1 (0.2)
Weekly physical activity	2 (0.41)

Table S2: Baseline covariates for mother-child pairs in the Faroe Island Cohort 5, stratified by whether or not the child completed a DXA scan at age 9.

Characteristic	No DXA Scan	DXA Scan	p-value*
n	107	368	
Sex = Male (%)	57 (53.3)	190 (51.6)	0.850
Maternal education (%)			0.047
Low	42 (39.3)	117 (31.8)	
Medium	18 (16.8)	105 (28.5)	
High	47 (43.9)	146 (39.7)	
Maternal smoking = No (%)	93 (86.9)	308 (83.7)	0.511
Parity = Multiparous (%)	72 (67.3)	262 (71.2)	0.510
Maternal BMI (median [IQR])	23.61 [21.67, 25.56]	23.74 [21.11, 26.10]	0.984

* p-values are for chi-square tests with continuity correction (categorical) or Wilcoxon rank-sum tests (non-normal continuous) tests when comparing population characteristics between males and females.

Table S3: Baseline covariates of study participants, stratified by sex

Characteristic	Female	Male	p-value*
n	177	189	
Maternal education (%)			0.238
Low	64 (36.2)	53 (28.0)	
Medium	48 (27.1)	55 (29.1)	
High	65 (36.7)	81 (42.9)	
Maternal smoking = No (%)	145 (81.9)	161 (85.2)	0.483
Parity = Multiparous (%)	119 (67.2)	143 (75.7)	0.095
Maternal BMI (mean (SD))	24.10 (4.09)	24.70 (4.82)	0.199
Weekly physical activity (%)			0.014
<=1	53 (29.9)	33 (17.5)	
2-3	85 (48.0)	114 (60.3)	
>=4	39 (22.0)	42 (22.2)	

* p-values are for chi-square tests with continuity correction (categorical) or Wilcoxon rank-sum tests (non-normal continuous) tests when comparing population characteristics between males and females.

Table S4: Baseline covariates of study participants, stratified by the time point of PFAS exposure measurement

Characteristic	Birth	18 months	5 years	9 years	p-value*
n	359	276	289	344	
Sex = Male (%)	185 (51.5)	141 (51.1)	148 (51.2)	179 (52.0)	0.995
Maternal education (%)					0.967
Low	114 (31.8)	84 (30.4)	86 (29.8)	107 (31.1)	
Medium	100 (27.9)	76 (27.5)	75 (26.0)	98 (28.5)	
High	145 (40.4)	116 (42.0)	128 (44.3)	139 (40.4)	
Maternal smoking = No (%)	301 (83.8)	230 (83.3)	241 (83.4)	289 (84.0)	0.994
Parity = Multiparous (%)	257 (71.6)	196 (71.0)	213 (73.7)	252 (73.3)	0.860
Maternal BMI (kg/m^2) (mean (SD))	24.39 (4.48)	24.60 (4.61)	24.66 (4.61)	24.47 (4.51)	0.874
Weekly physical activity, hours (%)					0.972
<=1	83 (23.1)	59 (21.4)	70 (24.2)	80 (23.3)	
2-3	196 (54.6)	154 (55.8)	149 (51.6)	185 (53.8)	
>=4	80 (22.3)	63 (22.8)	70 (24.2)	79 (23.0)	

* p-values are for chi-square tests with continuity correction (categorical) or Wilcoxon rank-sum tests (non-normal continuous) tests when comparing population characteristics between males and females.

Table S5: PFAS concentrations (ng/ml) by visit

Exposure	Birth	18 months	5 years	9 years
PFHxS (median [IQR])	0.17 [0.13, 0.22]	0.23 [0.09, 0.40]	0.32 [0.24, 0.44]	0.26 [0.20, 0.33]
PFOS (median [IQR])	2.87 [2.16, 4.05]	6.89 [4.36, 10.01]	4.71 [3.52, 6.18]	3.10 [2.45, 4.33]
PFOA (median [IQR])	0.89 [0.62, 1.35]	2.73 [1.92, 4.46]	2.22 [1.78, 2.80]	1.43 [1.19, 1.72]
PFNA (median [IQR])	0.31 [0.25, 0.42]	0.96 [0.63, 1.40]	1.12 [0.80, 1.60]	0.63 [0.50, 0.86]
PFDA (median [IQR])	0.09 [0.07, 0.12]	0.28 [0.21, 0.40]	0.33 [0.24, 0.46]	0.23 [0.17, 0.31]

Table S6: PFAS concentrations at birth (ng/ml) by study covariates

Characteristic	Level	PFHxS	PFOS	PFOA	PFNA	PFDA
Sex	Female	0.17 [0.13, 0.22]	2.84 [2.09, 3.89]	0.92 [0.64, 1.40]	0.32 [0.24, 0.43]	0.09 [0.07, 0.12]
Sex	Male	0.18 [0.13, 0.22]	2.93 [2.19, 4.11]	0.88 [0.61, 1.26]	0.31 [0.26, 0.42]	0.10 [0.07, 0.12]
Sex	p-value*	0.300	0.300	0.442	0.565	0.027
Maternal education	Low	0.17 [0.12, 0.21]	3.07 [2.14, 4.09]	0.97 [0.67, 1.40]	0.30 [0.24, 0.41]	0.08 [0.06, 0.12]
Maternal education	Medium	0.17 [0.13, 0.22]	2.95 [2.23, 4.04]	0.93 [0.68, 1.36]	0.34 [0.25, 0.43]	0.10 [0.07, 0.12]
Maternal education	High	0.17 [0.13, 0.22]	2.80 [2.01, 4.03]	0.84 [0.55, 1.21]	0.32 [0.25, 0.43]	0.09 [0.07, 0.12]
Maternal education	p-value*	0.647	0.422	0.086	0.238	0.081
Maternal smoking	Yes	0.20 [0.14, 0.25]	3.11 [2.45, 4.05]	0.96 [0.74, 1.45]	0.31 [0.26, 0.44]	0.10 [0.07, 0.13]
Maternal smoking	No	0.17 [0.12, 0.22]	2.83 [2.11, 4.04]	0.86 [0.60, 1.30]	0.32 [0.24, 0.42]	0.09 [0.07, 0.12]
Maternal smoking	p-value*	0.014	0.189	0.048	0.484	0.205
Parity	Primiparous	0.19 [0.15, 0.27]	3.35 [2.44, 4.89]	1.36 [0.97, 1.96]	0.35 [0.28, 0.47]	0.10 [0.07, 0.13]
Parity	Multiparous	0.16 [0.12, 0.21]	2.75 [2.01, 3.84]	0.74 [0.55, 1.08]	0.30 [0.24, 0.40]	0.09 [0.07, 0.12]
Parity	p-value*	<0.001	<0.001	<0.001	0.004	0.410
Maternal BMI	Underweight	0.16 [0.12, 0.28]	2.16 [1.76, 2.55]	0.99 [0.52, 1.37]	0.34 [0.26, 0.41]	0.10 [0.08, 0.12]
Maternal BMI	Normal	0.16 [0.13, 0.22]	2.75 [2.02, 3.88]	0.87 [0.58, 1.28]	0.30 [0.23, 0.42]	0.09 [0.07, 0.12]
Maternal BMI	Overweight	0.19 [0.13, 0.23]	3.24 [2.36, 4.30]	0.93 [0.72, 1.31]	0.33 [0.27, 0.42]	0.09 [0.07, 0.12]
Maternal BMI	Obese	0.18 [0.13, 0.24]	3.41 [2.39, 4.81]	0.85 [0.66, 1.38]	0.34 [0.27, 0.54]	0.09 [0.06, 0.13]
Maternal BMI	p-value*	0.631	0.008	0.558	0.347	0.900
Weekly physical activity	<=1	0.16 [0.13, 0.22]	3.10 [2.15, 4.49]	0.79 [0.56, 1.20]	0.32 [0.24, 0.49]	0.09 [0.07, 0.12]
Weekly physical activity	2-3	0.18 [0.13, 0.22]	2.86 [2.15, 4.04]	0.92 [0.63, 1.38]	0.32 [0.25, 0.42]	0.09 [0.07, 0.12]
Weekly physical activity	>=4	0.16 [0.12, 0.21]	2.81 [2.16, 3.70]	0.89 [0.63, 1.32]	0.30 [0.26, 0.39]	0.09 [0.07, 0.12]
Weekly physical activity	p-value*	0.484	0.464	0.394	0.720	0.834

* p-values are for Wilcoxon rank-sum tests (non-normal continuous) comparing PFAS distributions between population subgroups.

Table S7: Estimated total association between serum PFAS concentrations and aBMD at age 9, presented as the expected diference in aBMD for a doubling in serum PFAS.

Exposure	Effect Estimate (95% CI)	p-value
PFHxS, birth	0.1 (-0.017, 0.22)	0.091
PFOS, birth	0.015 (-0.1, 0.13)	0.8
PFOA, birth	0.026 (-0.078, 0.13)	0.63
PFNA, birth	-0.055 (-0.19, 0.079)	0.42
PFDA, birth	-0.049 (-0.19, 0.097)	0.51
PFHxS, 18 months	0.008 (-0.044, 0.06)	0.76
PFOS, 18 months	-0.044 (-0.15, 0.067)	0.44
PFOA, 18 months	-0.066 (-0.17, 0.04)	0.22
PFNA, 18 months	-0.09 (-0.2, 0.018)	0.1
PFDA, 18 months	-0.082 (-0.22, 0.053)	0.23
PFHxS, 5 years	-0.012 (-0.14, 0.11)	0.85
PFOS, 5 years	-0.1 (-0.26, 0.048)	0.18
PFOA, 5 years	-0.16 (-0.33, 0.0086)	0.063
PFNA, 5 years	-0.15 (-0.26, -0.039)	< 0.01
PFDA, 5 years	-0.07 (-0.17, 0.034)	0.19
PFHxS, 9 years	0.0037 (-0.15, 0.16)	0.96
PFOS, 9 years	-0.056 (-0.19, 0.075)	0.4
PFOA, 9 years	-0.14 (-0.36, 0.071)	0.19
PFNA, 9 years	-0.035 (-0.18, 0.11)	0.63
PFDA, 9 years	-0.012 (-0.15, 0.12)	0.86

Table S8: Estimated ADE and ACME from causal mediation models.

Exposure	Mediator	Effect	Effect Estimate (95% CI)	p-value
PFOS, 18 months	Total Fat Mass	ACME	-0.1 (-0.19, -0.023)	< 0.01
PFOS, 18 months	Total Fat Mass	ADE	0.063 (-0.078, 0.21)	0.4
PFOS, 18 months	Total Fat Mass	Total Effect	-0.038 (-0.21, 0.14)	0.73
PFOS, 18 months	Total Fat Mass	Prop. Mediated	2.7 (-13, 14)	0.72
PFOA, 18 months	Total Fat Mass	ACME	-0.1 (-0.19, -0.029)	0.012
PFOA, 18 months	Total Fat Mass	ADE	0.0066 (-0.13, 0.15)	0.87
PFOA, 18 months	Total Fat Mass	Total Effect	-0.097 (-0.25, 0.072)	0.27
PFOA, 18 months	Total Fat Mass	Prop. Mediated	1.1 (-6.1, 11)	0.26
PFOA, 18 months	BMI	ACME	-0.1 (-0.19, -0.0069)	0.038
PFOA, 18 months	BMI	ADE	0.0044 (-0.13, 0.13)	0.93
PFOA, 18 months	BMI	Total Effect	-0.096 (-0.25, 0.057)	0.28
PFOA, 18 months	BMI	Prop. Mediated	1 (-6, 6.2)	0.26
PFOA, 5 years	Total Fat Mass	ACME	-0.2 (-0.34, -0.064)	< 0.01
PFOA, 5 years	Total Fat Mass	ADE	-0.037 (-0.28, 0.22)	0.79
PFOA, 5 years	Total Fat Mass	Total Effect	-0.23 (-0.49, 0.046)	0.12
PFOA, 5 years	Total Fat Mass	Prop. Mediated	0.84 (-4, 4.2)	0.12
PFOA, 5 years	BMI	ACME	-0.21 (-0.37, -0.064)	< 0.01
PFOA, 5 years	BMI	ADE	-0.022 (-0.23, 0.2)	0.82
PFOA, 5 years	BMI	Total Effect	-0.23 (-0.49, 0.05)	0.098
PFOA, 5 years	BMI	Prop. Mediated	0.91 (-1.6, 5.4)	0.09
PFNA, 18 months	Total Fat Mass	ACME	-0.13 (-0.21, -0.051)	< 0.01
PFNA, 18 months	Total Fat Mass	ADE	0.014 (-0.14, 0.16)	0.83
PFNA, 18 months	Total Fat Mass	Total Effect	-0.11 (-0.28, 0.055)	0.19
PFNA, 18 months	Total Fat Mass	Prop. Mediated	1.1 (-5.8, 7.7)	0.19
PFNA, 18 months	BMI	ACME	-0.13 (-0.23, -0.044)	< 0.01
PFNA, 18 months	BMI	ADE	0.011 (-0.11, 0.15)	0.86
PFNA, 18 months	BMI	Total Effect	-0.12 (-0.29, 0.048)	0.16
PFNA, 18 months	BMI	Prop. Mediated	1.1 (-6.5, 11)	0.16
PFNA, 18 months	BMI (age 5 years)	ACME	-0.096 (-0.18, -0.019)	< 0.01
PFNA, 18 months	BMI (age 5 years)	ADE	-0.1 (-0.23, 0.043)	0.16
PFNA, 18 months	BMI (age 5 years)	Total Effect	-0.2 (-0.36, -0.027)	0.024
PFNA, 18 months	BMI (age 5 years)	Prop. Mediated	0.49 (0.11, 1.6)	0.032
PFDA, 18 months	Total Fat Mass	ACME	-0.16 (-0.26, -0.066)	< 0.01

Exposure	Mediator	Effect	Effect Estimate (95% CI)	p-value
PFDA, 18 months	Total Fat Mass	ADE	0.038 (-0.17, 0.23)	0.68
PFDA, 18 months	Total Fat Mass	Total Effect	-0.12 (-0.33, 0.1)	0.32
PFDA, 18 months	Total Fat Mass	Prop. Mediated	1.3 (-10, 11)	0.31
PFDA, 18 months	BMI	ACME	-0.15 (-0.27, -0.032)	0.01
PFDA, 18 months	BMI	ADE	0.032 (-0.15, 0.21)	0.76
PFDA, 18 months	BMI	Total Effect	-0.12 (-0.35, 0.1)	0.32
PFDA, 18 months	BMI	Prop. Mediated	1.3 (-9.7, 8.2)	0.31
PFDA, 18 months	BMI (age 5 years)	ACME	-0.12 (-0.21, -0.018)	0.022
PFDA, 18 months	BMI (age 5 years)	ADE	-0.077 (-0.25, 0.12)	0.45
PFDA, 18 months	BMI (age 5 years)	Total Effect	-0.19 (-0.4, 0.049)	0.11
PFDA, 18 months	BMI (age 5 years)	Prop. Mediated	0.6 (-1.5, 2.6)	0.12

2 Supplemental Figures

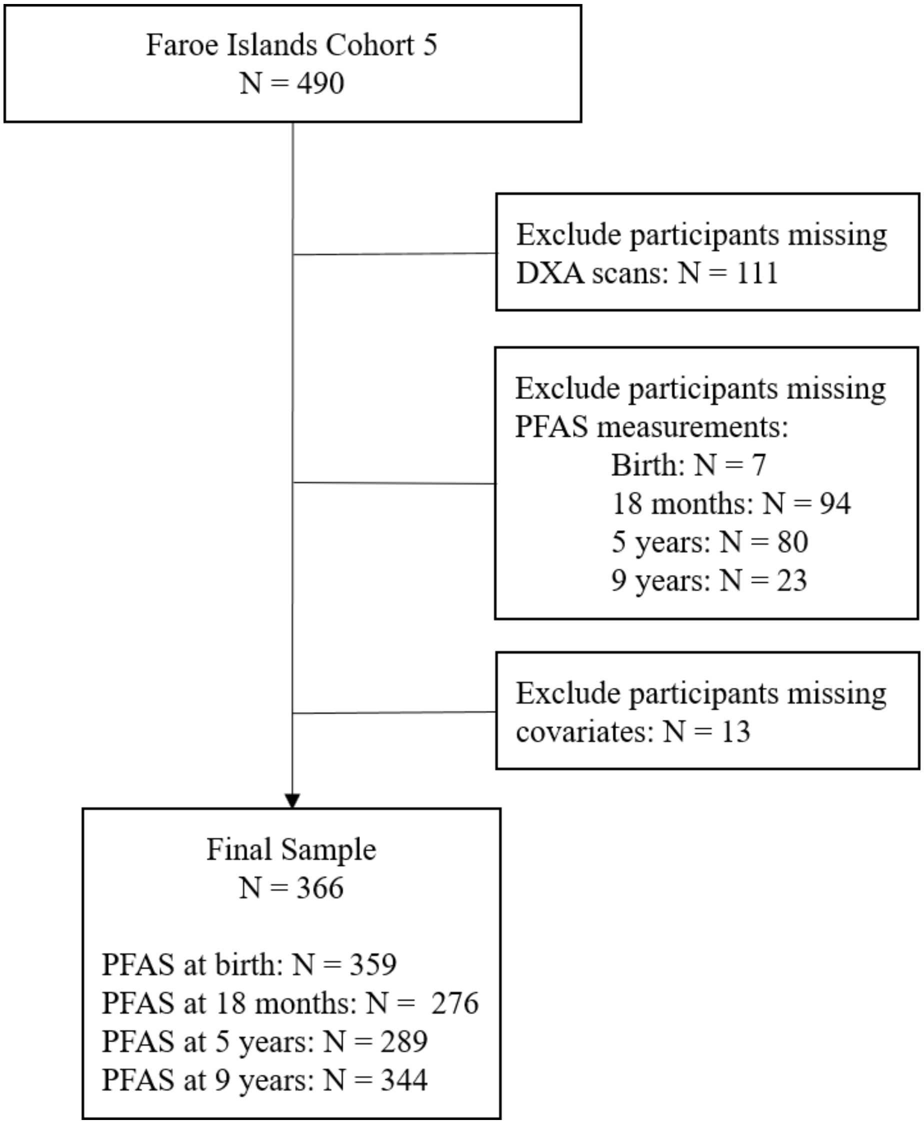

Figure S1: Study selection flow chart

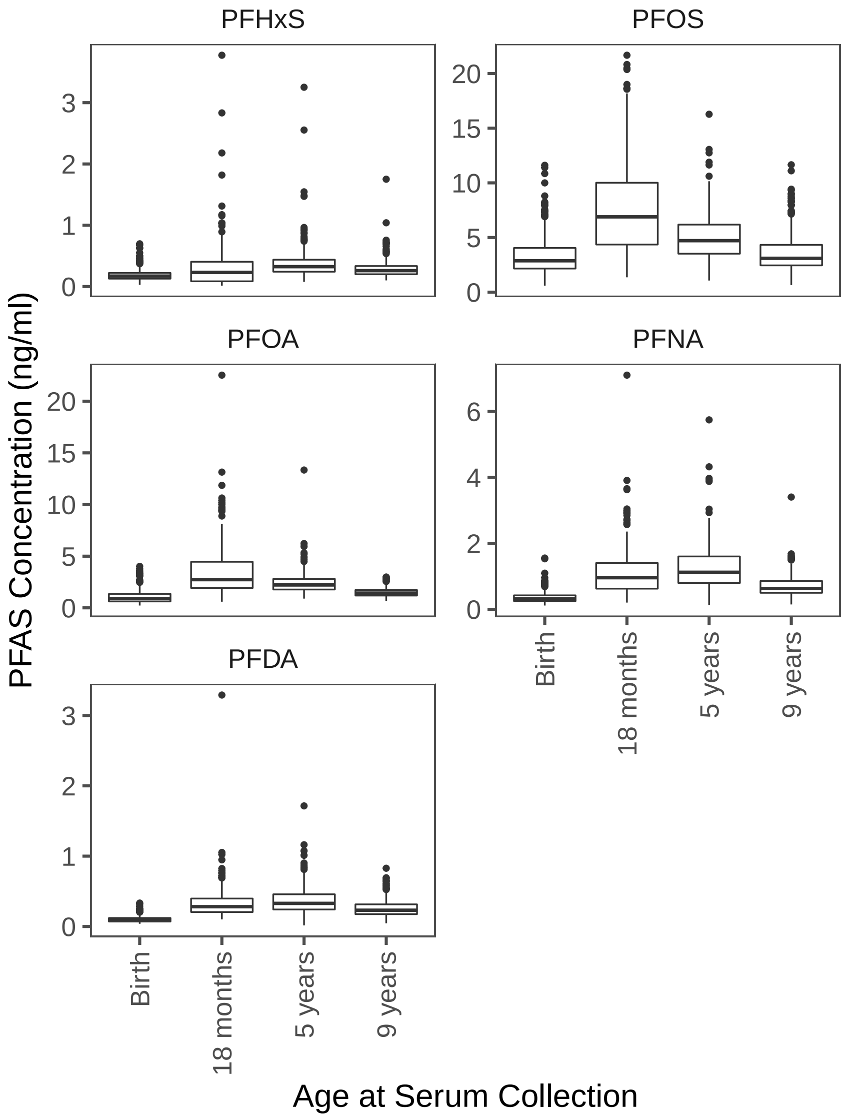

Figure S2: Distribution of serum PFAS concentrations by visit

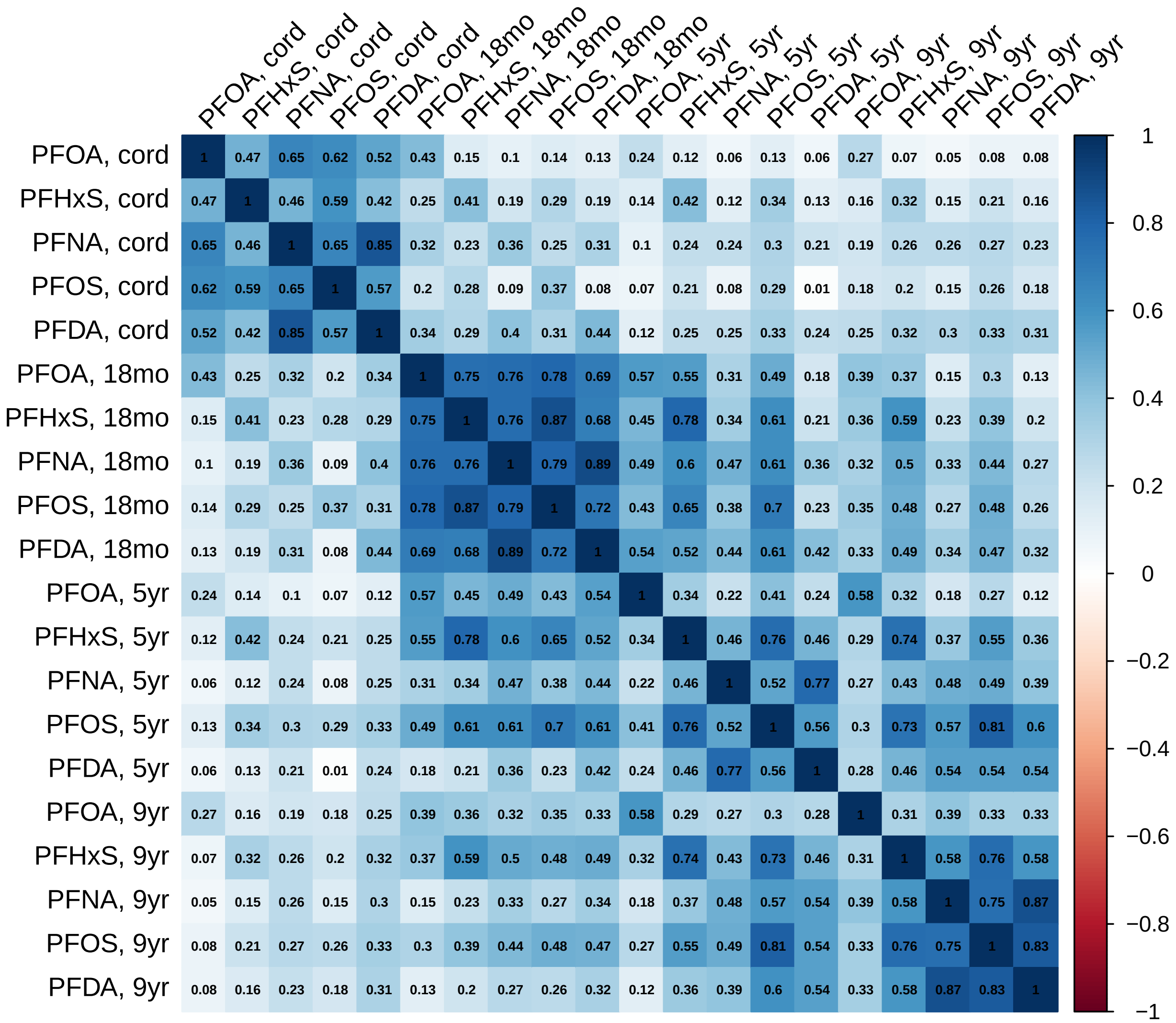

Figure S3: Correlation of serum PFAS concentrations

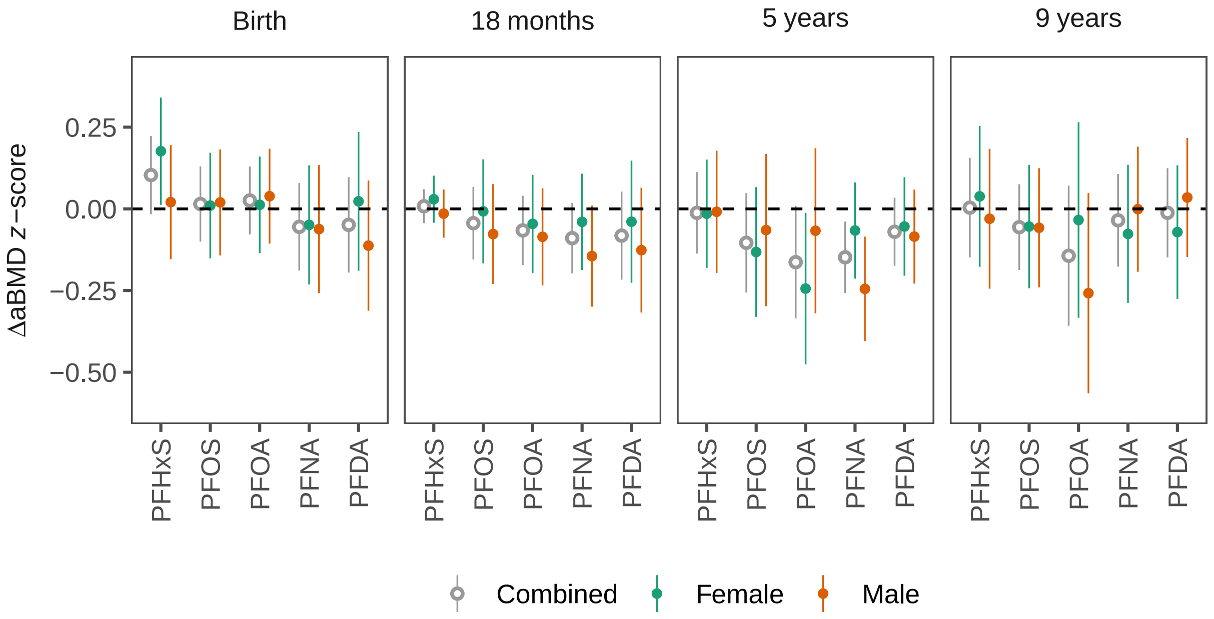

Figure S4: Associations between PFAS exposures measured at birth, 18 months, age 5 and age 9 and aBMD z-scores measured at age 9 by sex

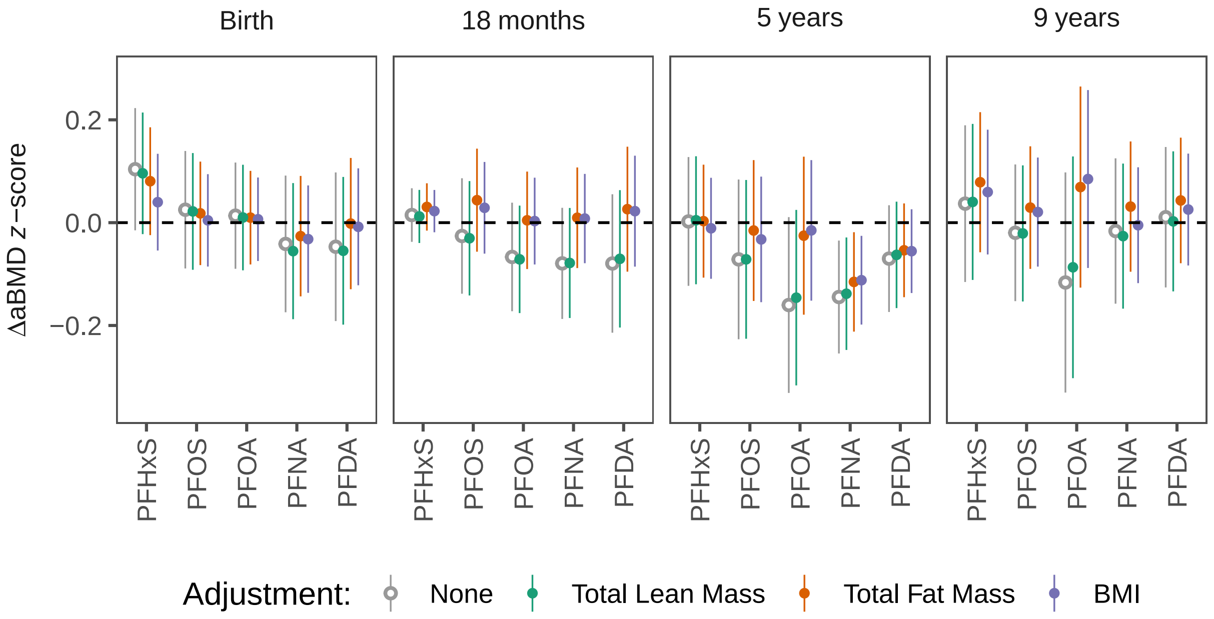

Figure S5: Direct and total associations between PFAS exposures measured at birth, 18 months, age 5, and age 9 and aBMD z-scores measured at age 9

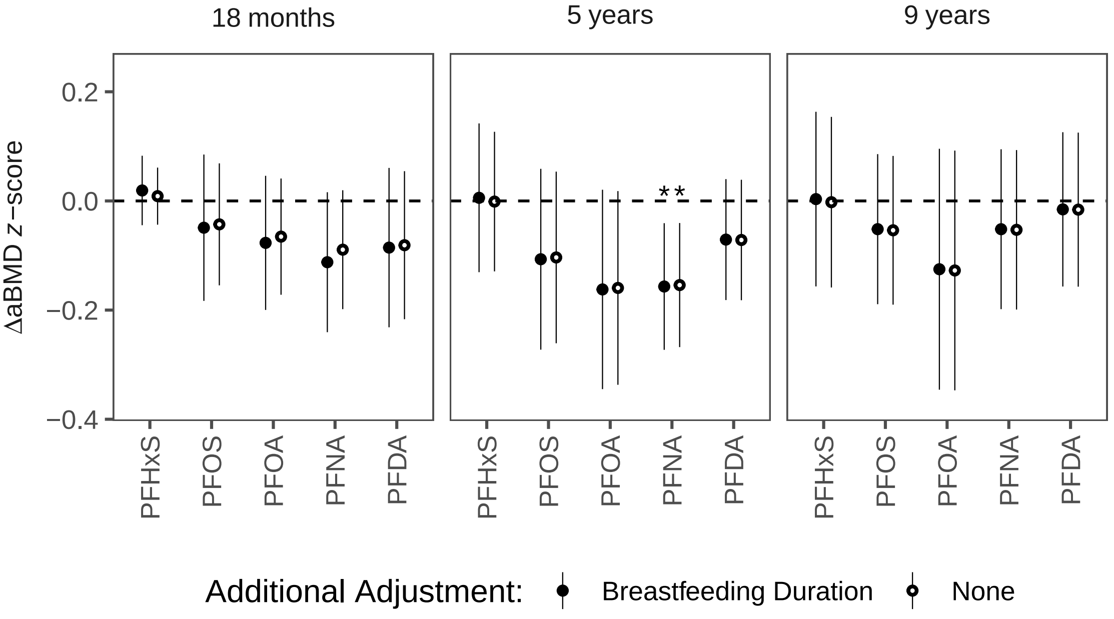

Figure S6: Associations between PFAS exposures measured at 18 months, and age 5 and 9 years and aBMD z-scores measured at age 9 years, with and without additional adjustment for duration of exclusive breastfeeding

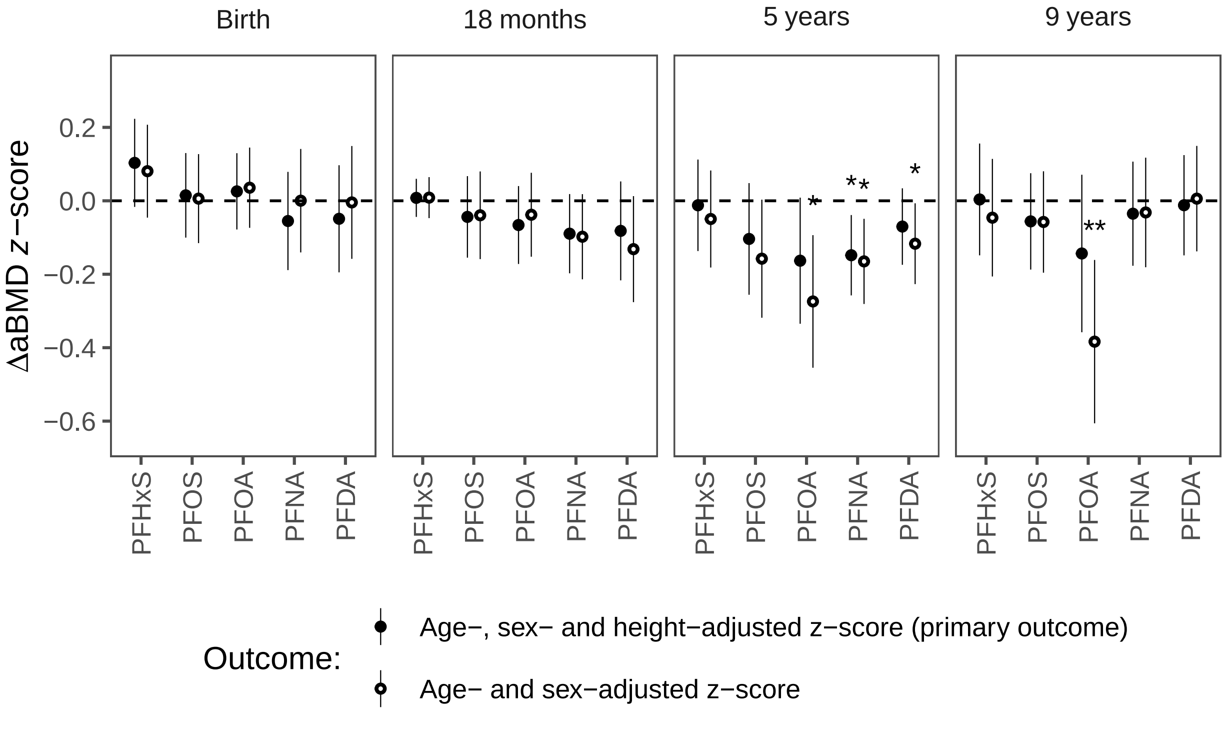

Figure S7: Comparison of associations between PFAS and age-, sex- and height-adjusted aBMD z-scores (primary outcome) vs. age- and sex-adjusted aBMD z-scores

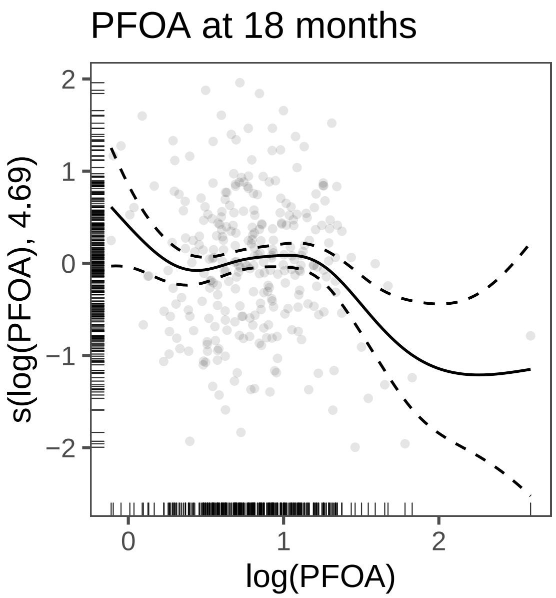

Figure S8: Estimated thin-plate splines for significant nonlinear PFAS terms

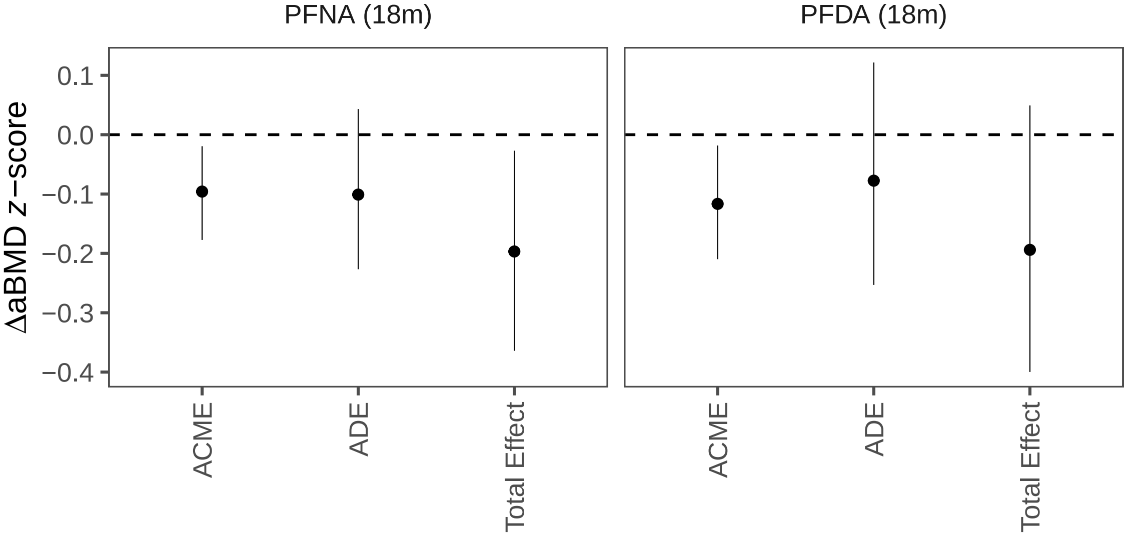

Figure S9: Mediation of the association between 18-month PFNA and PFDA concentrations and aBMD z-scores by BMI measured at age 5 years, limited to the 244 study participants who have 18-month PFAS measurements, 9-year DXA scans, complete covariate information and BMI values measured at age 5 years.
